# Thermoregulatory Responses and Performance of Dairy Calves Fed Different Amounts of Colostrum

**DOI:** 10.3390/ani11030703

**Published:** 2021-03-05

**Authors:** Fernanda Lavinia Moura Silva, Evangelina Miqueo, Marcos Donizete da Silva, Thaís Manzoni Torrezan, Nathalia Brito Rocha, Márcia Saladini Vieira Salles, Carla Maris Machado Bittar

**Affiliations:** 1 Department of Animal Science, Luiz de Queiroz College of Agriculture, University of São Paulo, Av. Pádua Dias, 11. Piracicaba, São Paulo 13.418-900, Brazil; fernandalmsilva@hotmail.com (F.L.M.S.); evangelina.miqueo@gmail.com (E.M.); silvamarcos@usp.br (M.D.d.S.); manzoni.thais@gmail.com (T.M.T.); n.britorocha@yahoo.com.br (N.B.R.); 2 Animal Science Institute (IZ/APTA), Av. Bandeirantes, 2419, Ribeirão Preto, São Paulo 14.030-670, Brazil; marcia.saladini@gmail.com

**Keywords:** Holstein calf, colostrum volume, thermogenesis

## Abstract

**Simple Summary:**

Colostrum is an important source of antibodies, nutrients, and energy for thermogenesis by the newborn calf. However, the amount of colostrum required for thermoregulatory responses and improved performance is not well established. This study evaluated newborn thermoregulatory responses during a cold challenge (10 °C) at 24 h of life and performance in the preweaning period for calves fed different volumes of colostrum. Newborn calves fed higher volumes of colostrum exhibited increased thermoregulatory responses, improved growth performance, and immunity.

**Abstract:**

This study investigates the newborn thermoregulatory responses to cold and the performance of calves fed different colostrum volumes. Thirty newborn Holstein calves were blocked by birth body weight (BW; 39.4 ± 6.5 kg) and fed different high-quality colostrum volumes: 10%, 15%, or 20% of BW, which was split and fed at 2 and 8 h after birth. At 24 h of life, calves were placed in a chamber at 10 °C for 150 min. Skin and rectal temperature (RT), heart and respiratory rate, and shivering were measured every 15 min. Blood samples were taken every 30 min. After the cold challenge, calves were housed in ambient temperature (26.8 ± 5.9 °C), with free access to water and concentrate and received 6 L/d of milk replacer. Feed intake, fecal score, and RT were recorded daily, until 56 d of age. Blood samples, BW, and body measures were taken weekly. During the cold challenge, prescapular temperature and total serum protein were greater for calves fed 15% or 20%. Leukocytes increased preweaning, presenting higher values for calves fed 20%. Even though there was a benefit for the calf submitted to cold stress on the first day of life, feeding higher volumes of colostrum resulted in no differences in performance during the preweaning phase. Nevertheless, calves fed a higher volume of colostrum (20% BW) presented increased immune responses during the preweaning phase.

## 1. Introduction

The calf acquires adequate passive immunity by ingestion and absorption of colostral immunoglobulins (Ig) soon after birth. The efficiency of Ig transfers across the gut epithelium is optimal during the first 4 h of life with a progressive decline 6 h after birth [1]. According to Osaka et al. [2], the apparent efficiency of absorption (AEA) of IgG declines by less than 0.3%/h from calving to 12 h after birth and 2.5%/h from 12 to at least 18 h after birth. Feeding calves with higher volumes of colostrum during the first hours of life promotes optimum growth, reduces veterinarian and medical costs, and increases future milk production [3,4,5].

In addition to its role in passive immunity transfer (PIT) and nutrient supply, colostrum feeding also helps to increase the tolerance to cold exposure [6]. The lower critical temperature for calves is suggested to be 13.4 °C [7]. Environmental conditions affect newborn calves’ survival and at a low critical temperature, morbidity and mortality may increase due to excessive heat loss [8]. Diesch et al. [9] reported lower rectal temperature for calves born during windy and wet weather and when ambient temperatures were <10 °C. Because of that, when an animal is acutely exposed to a lower critical temperature, it needs extra heat to compensate for the increased heat loss [10]. Several thermoregulatory mechanisms, such as body tissues metabolic rate, metabolism of brown adipose tissue (BAT), shivering, physical activity, and the feeding heat increment, are important for the adaptation to environmental conditions just after birth [11]. Thermogenic processes of the BAT include diet-induced thermogenesis and cold-induced non-shivering thermogenesis [12]. The amount of colostrum fed is positively correlated to heat production and body temperature due to the metabolic heat production represented by the energy cost associated with digestion, absorption, and metabolism of nutrients [13]. Colostrum supplies lactose, amino acids, and triglycerides, which constitute an excellent energy source (6.7 MJ/kg) for heat production both by diet-induced and non-shivering thermogenesis [14,15,16].

Godden [1] recommended feeding colostrum at 10% of BW within 6 h after birth. More recently, Lombard et al. [4] have reached a consensus recommendation of an additional feeding of 5% of BW within 6 h after the first feeding, resulting in a total of 15% of BW. However, recommendations for colostrum feeding have been based mainly on the success of PIT and decreased morbidity and mortality, regardless of the requirement to increase the calf’s tolerance to cold exposure.

We hypothesized that greater colostrum supply to newborn dairy calves would increase tolerance to cold exposure, leading to improved preweaning performance and health. The objective of this study was to investigate the newborn thermoregulatory responses to cold exposure during a 150 min cold challenge (10 °C) at 24 h of life and to evaluate the preweaning performance and health of dairy calves fed different amounts of colostrum.

## 2. Materials and Methods

### 2.1. Animals, Experimental Design, and Treatments

This study was conducted from November 2015 to February 2016 (26.8 ± 5.9 °C), at the Experimental Calf Facility of the “Luiz de Queiroz” College of Agriculture, University of São Paulo, Brazil. Thirty male Holstein calves (birth BW 39.4 ± 6.5 kg) were used. Immediately after birth, the calves were separated from their mothers and weighed.

A pool of first and second colostrum (mixed to reach a concentration of 60 mg of Ig/mL) was made before calves were born, frozen (−20 °C), and used in the study [1]. Colostrum quality was measured using a colostrometer (Suprivet, Divinópolis, MG, Brazil) at 22 °C since colostrometer data are better correlated than the Brix refractometer with IgG values [17]. Colostrum fat concentration was not measured because, according to our lab results, it has a low variation [18]. Colostrum was thawed in a water bath (50 °C) and fed to the newborn at 37 °C. Calves were blocked according to birth BW and were randomly distributed to one of the three different volumes of colostrum: low volume (10% BW of colostrum; n = 10), medium volume (15% BW of colostrum; n = 10), or high volume (20% BW of colostrum; n = 10). Within 2 h after birth, the calf was fed half the colostrum amount, and 6 h after the first feeding the second half was given. All calves were tube-fed to assure colostrum intake volume and time. Only calves born during the day were enrolled in the study because of the time window for the cold challenge.

### 2.2. Cold Challenge

**Figure 1:**
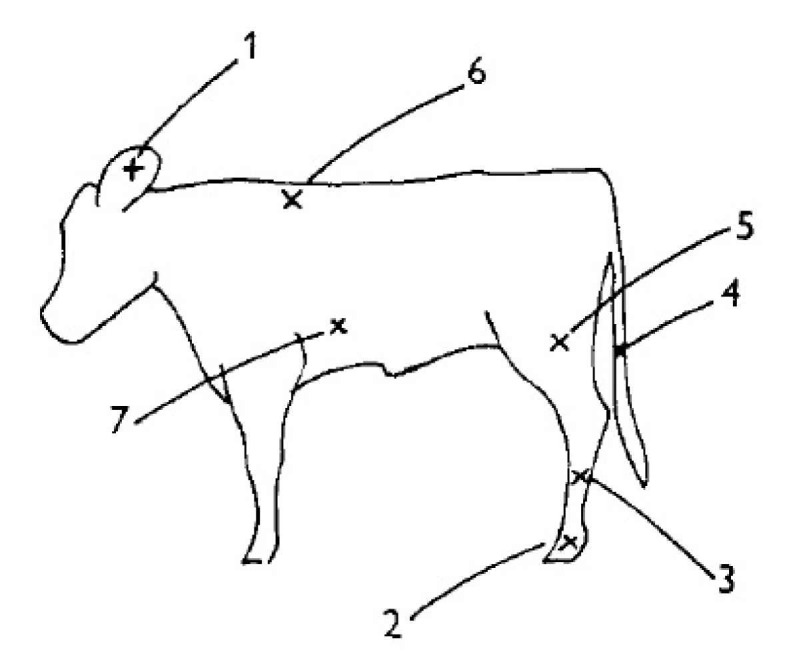
Skin surface measured throughout cold challenge. Ears (1), foot (2), shin (3), tail (4), muscular part of the thigh (5), prescapular (6), and thorax wall (7). Adapted from Gonzalez-Jimenez and Blaxter [19].

At exactly 24 h of life, each calf was fed 7.5% BW of whole milk (≈3 L) and then placed in an air circulating temperature-controlled chamber (2.0 × 1.80 × 2.10 m, Zero Grau Indústria de Refrigeração Ltd.a., Nova Santa Rosa, Brazil) at 10 °C, for 150 min. Rectal temperature, skin surface temperatures, heart rate, respiratory rate, and shivering were measured every 15 min starting at the moment the calf was placed into the chamber (time 0). The rectal temperature was taken using a digital thermometer and the skin surface temperatures were measured using an infrared thermometer (Instrutemp, São Paulo, Brazil), as described by Gonzalez-Jimenez and Blaxter [19]: prescapular, thorax wall, muscular part of the thigh, shin, foot, tail, and ears (Figure 1). Trichotomy was done in each surface area to improve temperature measures. A shivering score was applied, as suggested by Bellows and Lammoglia [20]: (1) no shivering; (2) moderate shivering of muscles in the back and legs; (3) intense shivering of muscles in back, legs and, face of the calf. Only two observers were involved in these measurements inside the cold chamber and they were not aware of the calf’s treatment.

### 2.3. Calf Performance

After the cold challenge, calves were individually housed outside (average temperature 26.8 ± 5.9 °C) in wood shelters distributed in a grassy field with free access to water and a pelleted commercial starter concentrate (21.71% CP; 4.46% Fat, 71.11% TDN; Agroceres Multimix, Rio Claro, SP, Brazil). Calves received 6 L/d of milk replacer diluted to 12.5% of solids (19.25% CP and 16.16% Fat on DM basis; Sucelac, Agroceres, Rio Claro, SP, Brazil), split into 2 feedings (0700 and 1700 h), until the eighth week of age when the study finished. The pelleted commercial starter was fed ad libitum every morning, and refusals were weighed to monitor daily intake. Milk replacer intake was also daily recorded. Animals were weighed weekly before morning milk supply using a mechanical scale (ICS-300, Coimma Ltd.a., Dracena, SP, Brazil), and withers height, heart girth, and hip-width were also measured. The withers height and hip-width were measured using a stick with a cm-scale (ruler), and the heart girth using a measuring tape. Every morning, fecal scores were recorded by a single observer using a scale from 0 to 3 (Available online: http://www.vetmed.wisc.edu/dms/fapm/fapmtools/8calf/calf_health_scoring_chart.pdf, accessed at 10 October 2015) according to the fluidity as (0) normal; (1) semi-formed, pasty; (2) loose, but stays on top of bedding; (3) watery, sifts through bedding. Weekly averages of scores were generated per calf for statistical analysis. Calves given a fecal score ≥ of 2 were considered to have diarrhea. When diarrhea was diagnosed, an oral electrolyte solution was offered, 2 h after milk feeding. Calves’ rectal temperature was measured daily, always after feeding. The animals were followed until 56 days of age, after that the gradual weaning process started. Data regarding the weaning period was not considered in the present study.

### 2.4. Blood Sampling and Analysis of Metabolites and Hormones

Blood samples were collected via jugular venipuncture into two vacuum tubes, one without anticoagulant and the other containing sodium fluoride and potassium EDTA (Vacuette of Brazil, Campinas, SP, Brazil). Sampling was done immediately before the cold challenge and every 30 min, for 150 min during the cold challenge, resulting in a total of 6-time points (0, 30, 60, 90, 120, 150 min). During the performance period, blood samples were taken weekly, 2 h after morning feeding. Samples were centrifuged at 2000× *g*, for 20 min at 4 °C to obtain plasma or serum, and were stored at −20 °C for subsequent analysis. Specific commercial enzymatic kits from LABTEST Diagnóstica S.A. (Lagoa Santa, MG, Brazil) were used to analyze total serum protein (TSP; Ref.: 99), albumin (Ref.: 19), glucose (Ref.: 85), lactate (Ref.: 116), and alkaline phosphatase (Ref.: 40). A commercial kit from Randox Laboratories (Life Sciences Ltd., Crumlin, UK) was used to analyze beta-hydroxybutyrate (BHB; Ref.: RB1007) and non-esterified fatty acids (NEFA; Ref.: FA115). All metabolites were measure in an automatic biochemistry system (SBA—200, CELM, Barueri, SP, Brazil). The concentration of globulin was calculated as the difference between total serum protein and albumin [21]. For the determination of insulin concentrations, samples were also taken at the 6-time points as described earlier. Insulin concentration was determined by a chemiluminescence immunoassay using the Immulite 1000 (Siemens Healthcare Diagnostics, Deerfield, IL, USA), with components of commercial kits (Diagnostic Products Corp., Los Angeles, CA, USA). The assay sensitivity was 2.0 μIU. Concentrations of T3 and T4 (analyzed at moment 0 and 120 min) and cortisol (analyzed at moment 0, 60, and 120 min after the calf was submitted to the cold challenge) were measured with commercial ELISA kits from Monobind Inc. (Lake Forest, CA, USA) and a microplate reader (EZ reader, Biochrom Ltd., Holliston, MA, USA). An aliquot of blood from the tube containing anticoagulant was used for hematocrit determination, after centrifugation (SPIN 1000–MICROSPIN) at 12,000× *g* for 10 min. Blood samples (0.02 mL) were diluted with 4 mL of Gower solution (12.5 g sodium sulfate and 33.3 mL glacial acetic acid in 100 mL on distilled water) for cell preservation. The dilution was pipetted into the Neubauer chamber and observed under a microscope (400 X, Bioval, PR, Brazil) for the total count of erythrocytes in µL. The mean corpuscular volume (MCV) was calculated considering the hematocrit and the total count of erythrocytes in µL. For the leukocytes count, blood samples (0.02 mL) were diluted with 0.4 mL of Turk solution (2 mL of acetic acid, 1 mL of gentian violet, 100 mL distilled water), pipetted into the Neubauer chamber, and observed under a microscope (400 X, Bioval, PR, Brazil).

### 2.5. Statistical Analysis

Data were analyzed using the PROC MIXED procedure of SAS version 9.3 (SAS/STAT, SAS Institute Inc., Cary, NC, USA) with models fitting a Gaussian distribution. Data were tested for normality of residuals using the GLM procedure. Homogeneity of variances followed Hovtest and Welsh methods, and normality of residuals was analyzed using the UNIVARIATE procedure of SAS following the Shapiro–Wilk method. Daily feed intake, fecal score, and rectal temperature during preweaning data were averaged for each week before statistical analysis. For data measured over time (hours or weeks), the analysis was performed as repeated measures, with the following statistical model:

*Yijk* = *μ* + *Ti* + *bj* + *eij* + *Ak* + *(bA)jk* + *(DA)ik* + *eijk*.

where, *Yijk* = dependent variable; *μ* = general average; *Ti* = fixed effect of Treatment; *bj* = random block effect; *eji* = residual error; *Ak* = fixed age (or time) effect; *(bA)jk* = random effect of block × age (or time) interaction; *(DA)ik* = fixed effect of the diet × age (or time) interaction, and *eijk* = residual error B. The covariance matrices “compound symmetry, heterogeneous compound symmetry, autoregressive, autoregressive heterogeneous, unstructured, banded, ante-dependence, variance components, toeplitz, and heterogeneous toeplitz” were tested and defined according to the lowest value obtained for “Akaike’s Information Criterion Corrected” (AICC) and the subject of the repeated measures used was animal (treatment). For all the response variables, the means were obtained through the LSMEANS command.

For data without repeated measures, the model used was:

*Yij* = *μ* + *Ti* + *Bj* + *Eij*

where *Yijk* = dependent variable; *μ* = Overall average; *Ti* was the treatment effect (colostrum volume); *Bj* was the random block effect; *Eij* = random experimental error. The repeated measures were analyzed according to the model:

*Yijk* = µ + *Ti* + *Bj* + *Ik* + *TIik* + *Eijk*

where *Yijk* was the response variable; *µ* was the overall mean; *Ti* was the treatment effect; *Bj* was the random block effect; *Ik* was the time or age effect; *TIik* was the effect of the interaction of treatment and time or age; and *Eijk* was the residual effect. The comparisons among the treatments were performed by the Tukey test when there was significance in the analysis of variance. When the F-test for interaction was significant, means were partitioned using the SLICE command in SAS. Significance was declared for values of *p* ≤ 0.05, whereas a tendency was defined as 0.10 ≥ *p* > 0.05.

## 3. Results

### 3.1. Cold Challenge

**Figure 2:**
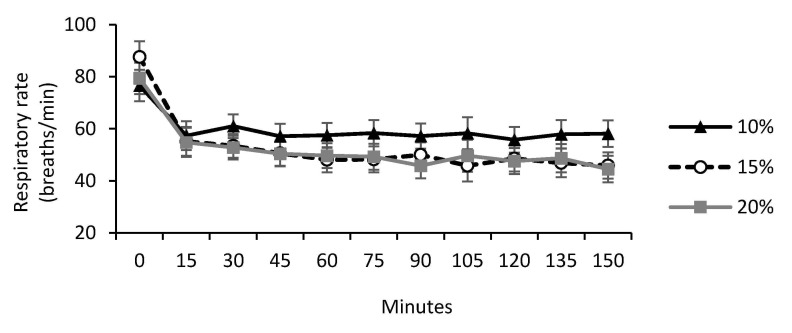
Respiratory rate of newborn dairy calves fed different volume of colostrum, across time effect during a cold challenge; 10% BW as colostrum (n = 10); 15% BW as colostrum (n = 10); 20% BW as colostrum (n = 10). Treatment by time effect interaction (*p* = 0.09).

There was no difference among treatments for heart rate (*p* < 0.88), but respiratory rate tended to be higher for the lowest colostrum feeding volume (*p* = 0.09, Table 1 and Figure 2). There was a tendency for colostrum volume to affect shivering during the cold challenge, with lower scores for calves fed 20% BW as compared to those fed 10% of BW, and calves fed 15% of BW being intermediate (*p* < 0.10; Table 1).

Colostrum volume intake tended to affect the rectal (*p* < 0.06) and the prescapular temperature (*p* < 0.09), with a lower temperature for calves fed 10% of BW as compared to 20% of BW, with no difference for those fed 15% of BW (Table 1). Skin temperature of the ear, thorax, thigh, shin, foot, and tail did not differ due to the colostrum feeding regimen. Rectal and skin temperature of the different evaluated body areas decreased as cold challenge time advanced, and drop abruptly right in the first 15 min of the cold challenge (*p* < 0.001; Supplementary Material).

The mean concentrations of TSP (*p* = 0.09), albumin (*p* = 0.08), and globulin (*p* = 0.03) were higher for calves fed 20% of BW as compared to those fed 10%, with no differences to calves fed 15% of BW (Table 1). The lactate concentration tended to be higher for calves fed 10 and 15% of BW compared to those fed 20% (*p* = 0.07). There was a time effect for all the selected blood metabolites evaluated during the cold challenge (*p* < 0.03; Table 1). While concentrations of TSP, globulin, lactate, NEFA, and alkaline phosphatase decreased, albumin and glucose increased as the time of the cold challenge advanced (Supplementary Material).

Mean values of insulin, cortisol, T3, and T4 during the cold challenge were not affected by colostrum feeding volume (Table 1). However, increased levels of insulin were observed as the time of cold exposure increased (*p* = 0.01), agreeing with the increased glucose concentrations (Supplementary Material).

### 3.2. Performance, Metabolism, and Health

**Figure 3:**
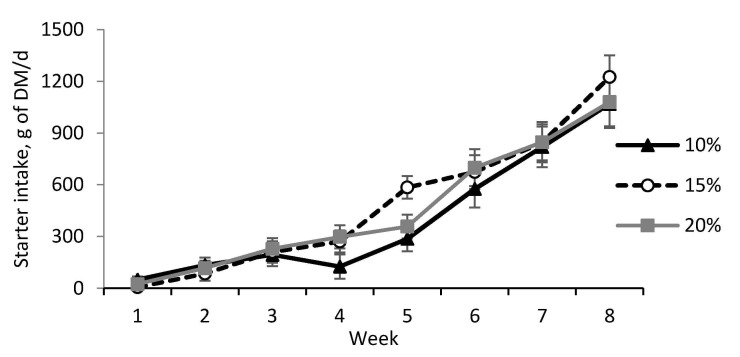
Preweaning starter intake of dairy calves previously fed different volumes of colostrum. 10% BW as colostrum (n = 10); 15% BW as colostrum (n = 10); 20% BW as colostrum (n = 10). Age effect (*p* < 0.001) and treatment by age interaction effect (*p* = 0.08).

Colostrum feeding volume did not affect preweaning performance (Table 2), except for a significant effect for heart girth (*p* < 0.05) and a tendency of the interaction of treatment and age for starter intake (*p* = 0.08). Heart girth was higher for calves fed 15 and 20% of BW compared to those fed 10%. Colostrum feeding volume resulted in a lower starter intake for calves fed 10% of BW at week 4 and higher values for calves fed 15% of BW at week 5 (Figure 3). Besides that, all performance parameters were affected by calves’ age (*p* < 0.01).

No differences were observed for selected blood metabolites evaluated during the preweaning phase as a function of colostrum feeding volume (*p* > 0.05), excepted for a tendency observed for BHB (*p* < 0.09) and NEFA (*p* < 0.08) concentrations (Table 3). Blood concentrations of BHB during the preweaning period tended to be higher for calves fed 15 and 20% of BW (*p* = 0.08). NEFA concentrations tended to be higher for calves fed colostrum as 10% as compared to 15% of BW, with no differences to calves fed 20% of BW (Table 3; Supplementary Material). However, all metabolites were affected by age (*p* < 0.001; Table 3).

**Figure 4:**
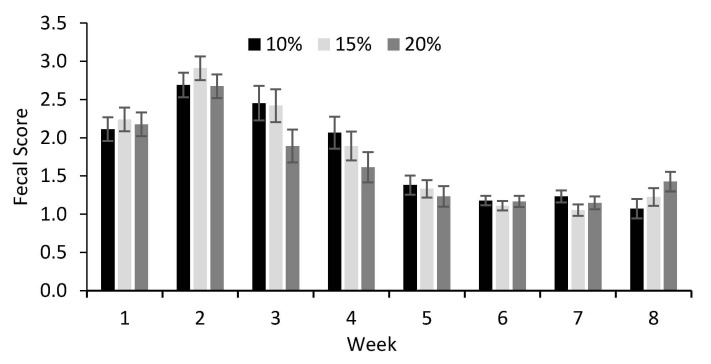
Average weekly fecal score of preweaning dairy calves previously fed different volumes of colostrum. 10% BW as colostrum (n = 10); 15% BW as colostrum (n = 10); 20% BW as colostrum (n = 10). Possible scores were (0) for firm feces, no diarrhea; (1) for soft feces, no diarrhea; (2) for mild diarrhea; and (3) for watery, severe diarrhea. Time effect (*p* < 0.001).

The fecal score evaluated at 24 h of life tended to be the lowest for calves fed colostrum as 10% of BW (*p* < 0.07; Table 4). No difference was observed among treatments for fecal score during the preweaning period. However, there was an age effect on fecal score (*p* < 0.001) with an increase during the second week of age for all treatments (Figure 4). No differences among treatments for rectal temperature and days with diarrhea during preweaning (Table 4). Mean values for hematocrit were not affected by treatment, but there was an age effect (*p* = 0.03, Table 4). Erythrocyte and MCV were also not affected by treatments (Table 4); however, erythrocyte count increased with age whereas MCV decreased. The average leukocyte count increased when the calves received 20% colostrum compared to the other treatments (*p* < 0.02).

## 4. Discussion

According to Davis and Drackley [7], as the environment temperature falls below the critical temperature, the body responds through physical and chemical means to reduce heat losses. The physical mechanisms come first, resulting in hair erection and peripheral vasoconstriction, reducing the body’s peripheral temperature [22]. The increased volume of colostrum probably provided more triglycerides for heat production by non-shivering thermogenesis, decreasing cold stress, and shivering responses. Intake of greater amounts of colostrum is positively related to the increased plasma triglycerides concentration [23], which are metabolized by the BAT to produce heat [24]. Thus, calves that received 15% and 20% of BW of colostrum presented lower shivering compared to calves that received 10%, probably due to increased heat production by the prescapular BAT, as a response of the increased fat intake. Our results suggest that the BAT present in the prescapular region produced more heat as the volume of colostrum increased, using substrates from colostrum for thermogenesis. Vermorel et al. [25] observed that heat production of newborn Holstein Friesian calves held at 10 °C increased by 13% between 1.5 and 2 h after the colostrum meal. The BAT generates heat by uncoupling oxidative metabolism from ATP synthesis in the mitochondria, with the release of heat [26]. Indeed, NEFA concentrations decreased with time of cold challenge, suggesting that this metabolite was used as a fuel by the BAT for heat production.

Respiratory rate could also have affected shivering amplitude because the inspiration of cold air causes an increase in rhythmic and tonic muscle activity, increasing shivering behavior [22], as observed in calves fed 10% of BW as colostrum. The respiratory rate tended to be higher for the lowest colostrum feeding volume (10%), probably due to an increase in depth and respiration rate (hyperventilation) caused by decreased rectal temperature. According to Conlon et al. [27], initial respiratory responses to cold weather are an increase in depth and respiration rate. However, for calves that received 15% or 20% BW of colostrum, respiratory rate tended to be lower at this moment, probably due to increased heat production by the BAT, which may have also resulted in increased rectal temperature.

The rectal temperature decreased as the time of cold challenge increased since lower temperatures induce a decrease in body core temperature [22]. Effects on body area temperature observed in the present study suggest that the blood supply to the peripheral areas was reduced as time in the cold challenge increased. Contrary to the current finding, some authors reported an increase in rectal temperature with time of cold exposure [20,28,29]. However, the authors observed those results placing calves in a 0 °C room for 140 min. According to Klingenspor et al. [30], the mechanism of increased temperature involves skin thermoreceptors activated by cold sensation, resulting in heat production. Thus, 10 °C was likely not cold enough to elicit this type of response from calves, despite the value for the lower critical temperature that increases thermogenesis in a one-day-old dairy calf being 13.4 °C [7]. Calves may be successfully be raised in areas with lower temperatures than that applied in the cold challenge, by the use of management tools. However, most producers in tropical and sub-tropical regions do not adopt those tools because the average temperature is usually high. Our data show that calves born in those regions, may be negatively affected by a cold challenge and that together with management tools, feeding more colostrum may benefit calves.

Knowles and Warriss [31] suggest that an initial stress response is the release of adrenaline and noradrenalin stimulating hepatic glycogenolysis, leading to increased plasma glucose levels. The increased concentrations of glucose may also have occurred due to the milk feeding right before the cold challenge, since postprandial plasma glucose increases after intake [32]. Increased levels of insulin were observed as the time of cold exposure increased, agreeing with the increased glucose concentrations. On the contrary, Bassett and Alexander [33] observed that during cold exposure insulin declined, reducing glucose uptake by peripheral tissues which may be used by central nervous tissue. According to Gruber et al. [34], more stressed behavior is also associated with increased lactate. Calves fed smaller amounts of colostrum in the present study showed greater concentrations of lactate, probably due to higher cold stress metabolic effects, as the higher respiratory rate. Under hypoxia, the anaerobic glucose metabolism is enhanced and, thus, a large amount of lactate is produced, which serve as the substrates for hepatic gluconeogenesis [35]. Additionally, concentrations of lactate are positively related to shivering [22], which corroborates to our results in this study.

TSP and globulin increased concentrations due to their positive correlation with colostrum ingestion [1]. Unfortunately, we have not determined the serum IgG concentration of calves. However, PIT analyzed by total globulin is statistically associated with that analyzed by IgG, being the globulins able to estimate serum IgG concentrations [36]. Thus, calves that received 15% or 20% BW of colostrum had higher concentrations of IgG, both at the beginning and during the cold challenge. If the more recent recommendation of the TSP cut point is applied [4], we could consider the PIT to be excellent since all calves present TSP > 6.2 g/dL. That was unexpected since feeding only 10% of BW as colostrum to the newborn could increase the proportion of calves with lower PST concentrations.

In contrast to our data, other authors have reported that cortisol concentrations were affected by time during cold stress, reaching peak concentration after approximately 10 to 20 min of cold exposure, and returning to initial concentrations after 80 min of exposure [20,28,29]. However, in the present study, cortisol was evaluated immediately before and then 60 min after cold exposure, probably too late to observe treatment or time effect. Cold stimulus after birth has been shown to increase T3 and T4 concentrations [26]. Stojić et al. [37] reported that different amounts of colostrum consumed by newborn calves had a minor effect on plasma concentrations of T3, T4, and cortisol. Cortisol stimulated by cold stress enhances the maturation of the thyroid axis leading to increased thyroid hormone levels and conversion of T4 to T3 in the BAT [16]. An effect according to the time of cold exposure was expected.

According to Hammon et al. [6], colostrum intake stimulates maturation and function of the gastrointestinal tract (GIT), mainly because of the presence of several growth factors. These compounds enhance digestive enzyme production and absorption capacity of nutrients [38], besides the effects on health. Because of those effects, feeding higher volumes of colostrum may stimulate the starter intake; however, that was not observed in the present study except at weeks 4 and 5. In the present study, because TSP at 24 h after birth increased with colostrum feeding volume, differences in BW and ADG were expected. However, there may be a TSP or an IgG concentration as a plateau for observing long-term effects on performance as a response to additional colostrum feeding.

The solid diet starts the rumen development through the conversion of butyrate to BHB by the ruminal wall, indicating initial rumen metabolic function [39]. Whereas circulating concentrations of BHB are highly correlated with concentrate intake, concentrations of NEFA indicates greater mobilization of fat due to low nutrient intake [40]. Therefore, in the current study, the tendency to increase starter intake at weeks 4 and 5 probably was sufficient to promote an effect on metabolites’ mean concentrations among treatments, leading to not only a tendency to increased BHB, but also a decreased NEFA concentration, as colostrum intake was higher.

Fecal score evaluated at 24 h of life tended to be higher for calves fed colostrum as 15% and 20% of BW, potentially due to decreased curd formation by abomasal enzymes dilution and consequently modification on the abomasal emptying pattern. Miyazaki et al. [41] suggest that calves exhibiting incomplete and no curd formation may be unable to absorb colostrum contents efficiently. In addition, the higher intake of fat through higher amounts of colostrum may cause intestinal epithelium saturation, leading to greater colostrum losses through feces. Thus, increasing the volume of colostrum fed may increase serum IgG only up to a point, after which some IgG losses may happen [42,43].

No difference was observed for a fecal score during the preweaning period. Indeed, there were no differences among treatments for rectal temperature and days with diarrhea during preweaning, suggesting adequate immune passive transfer for all treatments, even in the lowest volume fed group (10% of BW), which were adequate for PIT. Thus, the adequate volume of colostrum with the good quality provided for all treatments right after birth may have allowed calves to grow healthy in the same way.

Increased leukocyte count is usually related to innate responses to disease, especially in association with inflammatory processes and possibly at stress [44]. However, regardless of the colostrum feeding regimen, calves have grown healthy at a similar rate. In the present study, the mean leukocyte cell count was within the reference intervals for preweaning health dairy calves [45]. Thereby, the increased concentration of leukocytes in calves fed a higher volume of colostrum could be due to increased intake of colostral leukocytes, which may elicit a leukocyte response in calves, stimulating the development of neonatal immune responses [46]. In addition to the great importance of colostral Ig for the passive immunity of neonatal calves, colostrum contains a large number of immunomodulatory peptides that may affect neonatal immune response [47].

## 5. Conclusions

Feeding higher volumes of colostrum had a positive effect on newborn calves’ thermoregulatory responses during the cold challenge. However, even though there was a benefit for the calf submitted to cold stress on the first day of life, feeding higher volumes of colostrum resulted in no differences in performance during the preweaning phase. Nevertheless, calves fed a higher volume of colostrum (20% of birth weight) presented increased leukocyte count suggesting improved immune responses during the preweaning phase.

## Figures and Tables

**Table 1:** Rectal and the skin surface temperature, heart and respiratory rates, shivering, and selected metabolites and blood hormones during the cold challenge of newborn calves fed different volumes of colostrum.

Item ^1^	Treatments ^2^			SEM	*p*-Value ^3^		
	10%	15%	20%		Treat	Time	Treat * Time
Heart rate *, beats/min	85.1	84.2	82.1	4.66	0.88	0.25	0.17
Respiratory rate *, breaths/min	59.6	52.8	52.1	4.58	0.45	<0.001	0.09
Shivering score *	2.2 ^a^	2.0 ^ab^	1.7 ^b^	0.19	0.10	<0.001	0.77
Rectal temperature *, °C	37.7 ^b^	38.1 ^a^	38.0 ^a^	0.08	0.06	<0.001	0.32
Skin Surface Temperature *, °C							
Ear	19.4	19.9	20.8	1.14	0.66	<0.001	0.72
Prescapular	30.1 ^b^	30.7 ^ab^	31.9 ^a^	0.6	0.09	0.11	0.54
Thorax wall	32.5	33.0	33.4	0.45	0.36	<0.001	0.83
Thigh	30.5	30.0	30.6	1.1	0.87	<0.001	0.97
Shin	26.0	27.8	27.8	0.93	0.23	<0.001	0.62
Foot	23.2	26.8	24.6	1.14	0.11	<0.001	0.11
Tail	17.4	22.5	20.9	1.18	0.18	<0.001	0.34
Selected Blood Metabolites **							
Total protein, g/dL	6.4 ^b^	6.7 ^ab^	7.1 ^a^	0.23	0.09	<0.001	0.86
Albumin, g/dL	2.5 ^b^	2.4 ^ab^	2.4 ^a^	0.05	0.08	0.01	0.48
Globulin, g/dL	3.86 ^b^	4.36 ^ab^	4.73 ^a^	0.22	0.03	<0.001	0.92
Glucose, mg/dL	125.6	127.1	141.3	8.51	0.34	<0.001	0.90
Lactate, mg/dL	46.7 ^a^	44.9 ^a^	34.6 ^b^	3.85	0.07	0.03	0.17
NEFA, mmol/L	0.5	0.6	0.5	0.05	0.20	<0.001	0.73
Alk. Phosphatase, UI	174.1	187.1	153.7	23.7	0.33	<0.001	1.00
**Blood hormones**							
Insulin **, μUI	3.8	4.7	4.0	0.68	0.61	0.01	0.71
Cortisol ^¥^, µg/dL	6.8	7.1	8.1	0.85	0.32	0.12	0.75
T3 ^§^, ng/dL	4.2	3.5	4.3	0.55	0.56	0.81	0.35
T4 ^§^, ng/dL	1.3	1.2	1.1	0.19	0.53	0.30	0.36

^1^ T3 = Triiodothyronine; T4 = thyroxine. ^2^ 10%, 15% e 20% = BW as colostrum. ^3^ *p*-value for treatment (Treat), Time, and treatment by time interaction (Treat*Time). * measured every 15 min, from time 0 to 150 min during the cold challenge. ** measured every 30 min, from time 0 to 150 min during the cold challenge. ^¥^ measured at 0, 60, and 120 min during the cold challenge. ^§^ measured at 0 and 120 min during the cold challenge. ^ab^ Means within a row with different superscript differs with *p* < 0.05.

**Table 2:** Growth and intake of dairy calves fed different volumes of colostrum at birth.

Item	Treatments ^1^			SEM	*p*-Value ^2^		
	10%	15%	20%		Treat	Age ^3^	Treat *Age
Starter intake, g DM/d	407.1	488.2	456.5	50.05	0.52	<0.001	0.08
Total intake, g DM/d	1098.0	1178.4	1156.2	77.66	0.65	<0.001	0.14
Body weight, kg	47.1	50.9	50.2	4.88	0.22	<0.001	0.73
Average gain, g/d	481.4	544.4	552.8	55.24	0.48	<0.001	0.42
Feed efficiency	0.4	0.4	0.5	0.03	0.20	<0.001	0.42
Heart girth, cm	84.1 ^b^	86.9 ^a^	86.6 ^a^	2.65	0.05	<0.001	0.10
Hip width, cm	22.9	23.6	23.3	0.69	0.18	<0.001	0.34
Withers height, cm	82.7	84.2	82.6	1.88	0.32	<0.001	0.94

^1^ 10%, 15% e 20% = BW as colostrum. ^2^ *p*-value for treatment (Treat), age, and treatment by age interaction (Treat*Age). ^3^ Measures were taken weekly. ^ab^ Means within a row with different superscript differs with *p* < 0.05.

**Table 3:** Blood metabolite concentrations of dairy calves fed different volumes of colostrum at birth.

Item ^1^	Treatments ^2^			SEM	*p*-Value ^3^		
	10%	15%	20%		Treat	Age ^4^	Treat *Age
Total protein, g/dL	5.4	5.5	5.6	0.09	0.32	<0.001	0.17
Albumin, g/dL	2.8	2.8	2.9	2.83	0.30	<0.001	0.52
Glucose, mg/dL	98.6	107.9	102.7	6.00	0.54	<0.001	0.95
Lactate, mg/dL	13.5	14.2	14.3	0.89	0.80	<0.001	0.30
BHB, mmol/L	0.07 ^b^	0.09 ^a^	0.09 ^a^	0.005	0.09	<0.001	0.50
NEFA, mmol/L	0.21 ^a^	0.16 ^b^	0.19 ^ab^	0.19	0.08	<0.001	0.49

^1^ BHB = beta-hydroxybutyrate; NEFA = Non esterified fatty acids. ^2^ 10%, 15% e 20% = BW as colostrum. ^3^ *p*-value for treatment (Treat), age, and treatment by age interaction (Treat *Age). ^4^ Calves were sample every week. ^ab^ Means within a row with different superscript differs with *p* < 0.05.

**Table 4:** Fecal score, diarrhea occurrence, rectal temperature, hematocrit, erythrocytes, and leukocytes of dairy calves fed different volumes of colostrum at birth.

Item	Treatments ^1^			SEM	*p*-Value ^2^		
	10%	15%	20%		Treat	Age	Treat * Age
**Fecal score**							
24 h after birth	1.0 ^b^	1.5 ^a^	1.5 ^a^	0.18	0.07	-	-
Preweaning	1.8	1.8	1.7	0.07	0.42	<0.001	0.46
Diarrhea days	10.3	12.4	9.3	2.08	0.28	-	-
Rectal temperature, °C	38.3	38.3	38.3	0.06	0.87	<0.001	0.27
Hematocrit, %	20.1	20.0	20.5	0.95	0.74	0.03	0.96
Erythrocytes, 10^6^/µL	7.1	7.2	6.9	0.27	0.65	<0.001	0.23
MCV ^3^, µm	29.8	28.4	30.9	0.96	0.17	<0.001	0.51
Leukocytes, 10^3^/µL	6.3 ^b^	6.3 ^b^	7.4 ^a^	0.37	0.02	0.02	0.85

^1^ 10%, 15% e 20% = BW as colostrum. ^2^ *p* value for treatment (Treat), age, and treatment by age interaction (Treat*Age). ^3^ MCV = mean corpuscular volume. ^ab^ Means within a row with different superscript differs with *p* < 0.05.

## Data Availability

The data presented in this study are available on request from the corresponding author. The data are not publicly available due to restrictions by the research group.

## Supplementary Materials

The following are available online at https://www.mdpi.com/2076-2615/11/3/703/s1, Figure S1: Rectal and the skin surface temperature across time during a cold challenge of newborn dairy calves fed different volume of colostrum. Figure S2: Shivering across time, during a cold challenge of newborn dairy calves fed different volume of colostrum. Figure S3: Blood metabolites of newborn dairy calves fed different volume of colostrum, across time effect during a cold challenge. Figure S4: Preweaning beta-hydroxybutyrate concentrations of dairy calves fed different volumes of colostrum at birth.

## References

[1] Godden S. (2008). Colostrum management for dairy calves. Vet. Clin. N. Am. Food Anim. Pract..

[2] Osaka I., Matsui Y., Terada F. (2014). Effect of the mass of immunoglobulin (Ig)G intake and age at first colostrum feeding on serum IgG concentration in Holstein calves. J. Dairy Sci..

[3] Hammon H.M., Schiessler G., Nussbaum A., Blum J.W. (2002). Feed intake patterns, growth performance, and metabolic and endocrine traits in calves fed unlimited amounts of colostrum and milk by automate, starting in the neonatal period. J. Dairy Sci..

[4] Lombard J., Urie N.J., Garry F., Godden S., Quigley J.D., Earleywine T., McGuirk S., Moore D., Branan M., Chamorro M. (2020). Consensus recommendations on calf- and herd-level passive immunity in dairy calves in the United States. J. Dairy Sci..

[5] Faber S.N., Faber N.E., Mccauley T.C., Ax R.L. (2005). Case Study: Effects of colostrum ingestion on lactational performance. Prof. Anim. Sci..

[6] Hammon H.M., Steinhoff-Wagner J., Schönhusen U., Metges C.C., Blum J.W. (2012). Energy metabolism in the newborn farm animal with emphasis on the calf: Endocrine changes and responses to milk-born and systemic hormones. Domest. Anim. Endocrinol..

[7] Davis C.L., Drackley J.K. (1998). The Development, Nutrition, and Management of the Young Calf.

[8] Bellows R.A. Factors affecting calf survival. Proceedings of the 15th Range Beef Cow Symposium.

[9] Diesch T., Mellor D., Stafford K., Ward R. (2004). The physiological and physical status of single calves at birth in a dairy herd in New Zealand. N. Z. Vet. J..

[10] Cannon B., Nedergaard J. (2004). Brown adipose tissue: Function and physiological significance. Physiol. Rev..

[11] Silva F.L.M., Bittar C.M.M. (2019). Thermogenesis and some rearing strategies of dairy calves at low temperature—A review. J. Appl. Anim. Res..

[12] Harper M.-E., Antoniou A., Bevilacqua L., Bezaire V., Monemdjou S. (2002). Cellular energy expenditure and the importance of uncoupling1. J. Anim. Sci..

[13] Herpin P., Louveau I., Damon M., Le Dividich J. (2005). Chapter 14 Environmental and hormonal regulation of energy metabolism in early development of the pig. Biology of Growing Animals.

[14] Vermorel M., Dardillat C., Vernet J., Demigne C. (1983). Energy metabolism and thermoregulation in the newborn calf. Ann. Rech. Vet..

[15] Himms-Hagen J. (1990). Brown adipose tissue thermogenesis: Interdisciplinary studies. FASEB J..

[16] Danijela K. (2015). Endocrine and metabolic adaptations of calves to extra-uterine life. Acta Vet. Brno..

[17] Bartier A.L., Windeyer M.C., Doepel L. (2015). Evaluation of on-farm tools for colostrum quality measurement. J. Dairy Sci..

[18] Dos Santos G., da Silva J.T., da Rocha Santos F.H., Bittar C.M.M. (2017). Nutritional and microbiological quality of bovine colostrum samples in Brazil. Rev. Bras. Zootec..

[19] Gonzalez-Jimenez E., Blaxter K.L. (1962). The metabolism and thermal regulation of calves in the first month of life. Br. J. Nutr..

[20] Bellows R.A.A., Lammoglia M.A.A. (2000). Effects of severity of dystocia on cold tolerance and serum concentrations of glucose and cortisol in neonatal beef calves. Theriogenology.

[21] Jolles S., Borrell R., Zouwail S., Heaps A., Sharp H., Moody M., Selwood C., Williams P., Phillips C., Hood K. (2014). Calculated globulin (CG) as a screening test for antibody deficiency. Clin. Exp. Immunol..

[22] Pozos R.S., Danzl D.F., Pandolf K.B., Burr R.E. (2001). Human Physiological responses to cold stress and hypothermia. Medical Aspects of Harsh Environments.

[23] Rauprich A.B.E., Hammon H.M., Blum J.W. (2000). Influence of feeding different amounts of first colostrum on metabolic, endocrine, and health status and on growth performance in neonatal calves. J. Anim. Sci..

[24] Himms-Hagen J. (1985). Brown adipose tissue metabolism and thermogenesis. Annu. Rev. Nutr..

[25] Vermorel M., Vernet J., Saido, Dardillat C., Demigne C. (1989). Energy metabolism and thermoregulation in the newborn calf; variations during the first day of life and differences between breeds. Can. J. Anim. Sci..

[26] Hillman N.H., Kallapur S.G., Jobe A.H. (2012). Physiology of transition from intrauterine to extrauterine Life. Clin. Perinatol..

[27] Conlon K.C., Rajkovich N.B., White-Newsome J.L., Larsen L., O’Neill M.S. (2011). Preventing cold-related morbidity and mortality in a changing climate. Maturitas.

[28] Lammoglia M.A., Bellows R.A., Grings E.E., Bergman J.W., Short R.E., MacNeil M.D. (1999). Effects of feeding beef females supplemental fat during gestation on cold tolerance in newborn calves. J. Anim. Sci..

[29] Lammoglia M.A., Bellows R.A., Grings E.E., Bergman J.W. (1999). Effects of prepartum supplementary fat and muscle hypertrophy genotype on cold tolerance in newborn calves. J. Anim. Sci..

[30] Klingenspor M., Fromme T., Bast A., Bolze F., Li Y., Maurer S., Schweizer S., Willershäuser M., Fromme T. (2017). Brown adipose tissue. Adipose Tissue Biology.

[31] Knowles T.G., Warriss P.D., Vogel K. (2014). Stress physiology of animals during transport. Livestock Handling and Transport.

[32] Omidi-Mirzaei H., Khorvash M., Ghorbani G.R., Moshiri B., Mirzaei M., Pezeshki A., Ghaffari M.H. (2015). Effects of the step-up/step-down and step-down milk feeding procedures on the performance, structural growth, and blood metabolites of Holstein dairy calves. J. Dairy Sci..

[33] Bassett J.M., Alexander G. (1971). Insulin, growth hormone and corticosteroids in neonatal lambs. Neonatology.

[34] Gruber S.L., Tatum J.D., Engle T.E., Chapman P.L., Belk K.E., Smith G.C. (2010). Relationships of behavioral and physiological symptoms of preslaughter stress to beef longissimus muscle tenderness1. J. Anim. Sci..

[35] Tao S., Dahl G.E. (2013). Invited review: Heat stress effects during late gestation on dry cows and their calves. J. Dairy Sci..

[36] Weaver D.M., Tyler J.W., VanMetre D.C., Hostetler D.E., Barrington G.M. (2000). Passive transfer of colostral immunoglobulins in Calves. J. Vet. Intern. Med..

[37] Velibor S., Anna N.-J., Gy H., Horea S., Dragan G., Danijela K. (2002). The plasma levels of triiodothyronine, thyroxine and cortisol in newborn calves. Acta Vet. Brno..

[38] Bach A. (2012). Ruminant nutrition symposium: Optimizing performance of the offspring: Nourishing and managing the dam and postnatal calf for optimal lactation, reproduction, and immunity1,2. J. Anim. Sci..

[39] Khan M.A., Bach A., Weary D.M., von Keyserlingk M.A.G. (2016). Invited review: Transitioning from milk to solid feed in dairy heifers. J. Dairy Sci..

[40] Abdelgadir I.E.O., Morrill J.L., Higgins J.J. (1996). Effect of roasted soybeans and corn on performance and ruminal and blood metabolites of dairy calves. J. Dairy Sci..

[41] Miyazaki T., Okada K., Miyazaki M. (2017). Short communication: Neonatal calves coagulate first-milking colostrum and produce a large curd for efficient absorption of immunoglobulins after first ingestion. J. Dairy Sci..

[42] Conneely M., Berry D.P., Murphy J.P., Lorenz I., Doherty M.L., Kennedy E. (2014). Effect of feeding colostrum at different volumes and subsequent number of transition milk feeds on the serum immunoglobulin G concentration and health status of dairy calves. J. Dairy Sci..

[43] Da Silva A.P., de Toledo A.F., Cezar A.M., Coelho M.G., Virginio Júnior G.F., Poczynek M., Silva M.D., Haines D.M., Campos M., Bittar C.M.M. (2020). Passive transfer and neonatal health in dairy calves receiving maternal colostrum and/or a colostrum replacer. Livest. Sci..

[44] Hulbert L.E., Moisá S.J. (2016). Stress, immunity, and the management of calves1. J. Dairy Sci..

[45] Jezek J., Nemec M., Staric J., Klinkon M. (2011). Age related changes and reference intervals of haematological variables in dairy calves. Bull. Vet. Inst. Puławy.

[46] Reber A.J., Hippen A.R., Hurley D.J. (2005). Effects of the ingestion of whole colostrum or cell-free colostrum on the capacity of leukocytes in newborn calves to stimulate or respond in one-way mixed leukocyte cultures. Am. J. Vet. Res..

[47] Hammon H.M., Steinhoff-Wagner J., Flor J., Schönhusen U., Metges C.C. (2013). Lactation biology symposium: Role of colostrum and colostrum components on glucose metabolism in neonatal calves1,2. J. Anim. Sci..

