# Supplementary material for: Thermoregulatory Responses and Performance of Dairy Calves Fed Different Amounts of Colostrum
Source: Animals (Basel). 2021 Mar 5;11(3):703. doi: 10.3390/ani11030703 (PMC8001325; doi:10.3390/ani11030703)
Supplement: Supplementary file 1 [file animals-11-00703-s001.pdf]

Supplementary Material

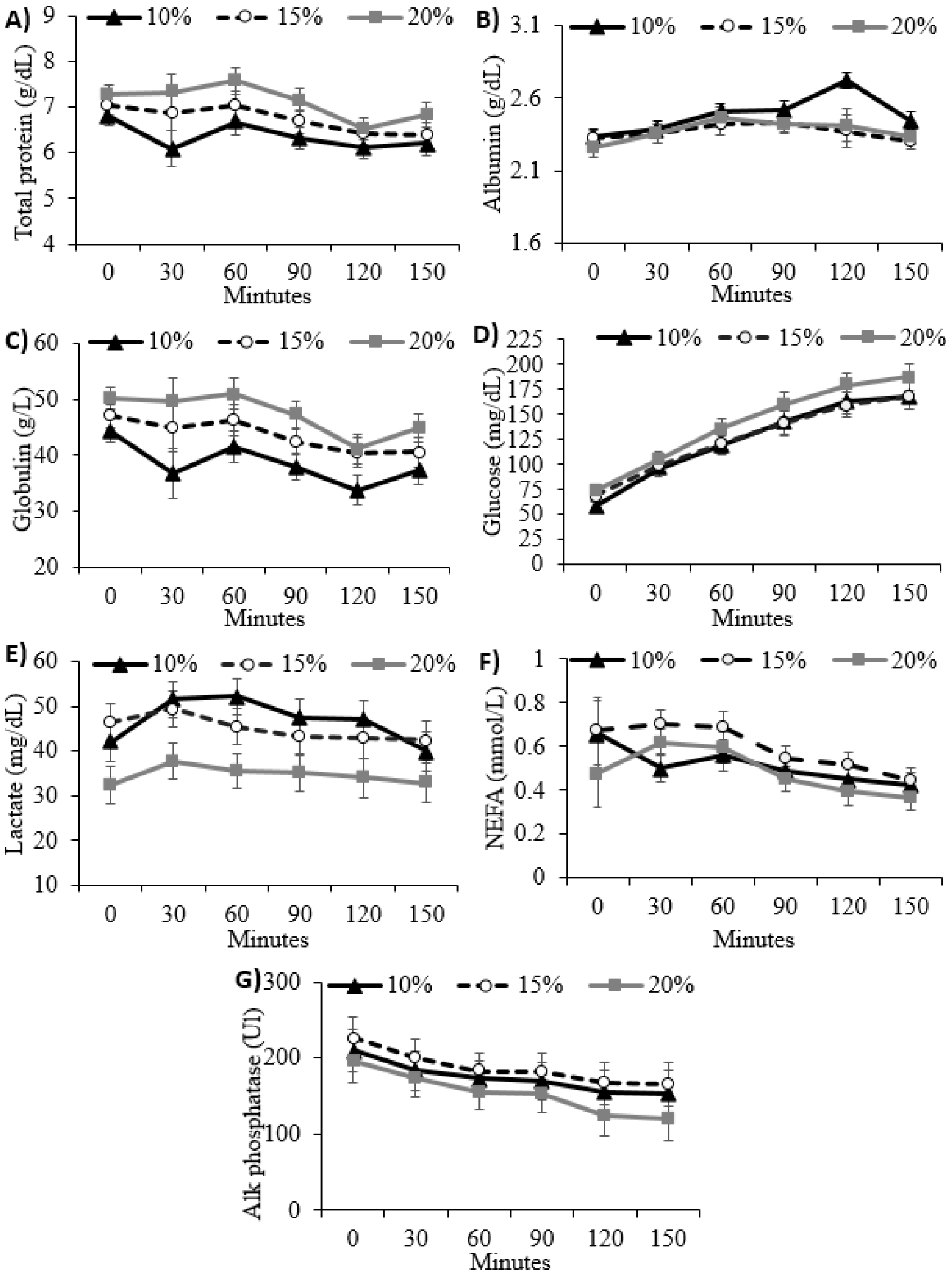

Figure S1 Rectal and the skin surface temperature across time during a cold challenge of newborn dairy calves fed different volume of colostrum. 10% BW as colostrum (n = 10); 15% BW as colostrum (n = 10); 20% BW as colostrum (n = 10). (A) Rectal temperature; Average (10% = 37.7[b], 15% = 38.1[a], 20% = 38.0[a]; SEM 0.08), treatment effect (P = 0.06), time effect (P < 0.001) and treatment by time effect interaction (P = 0.32). (B) Ear; Average (10% = 19.4,

15% = 19.9, 20% = 20.8; SEM 1.14), treatment effect (P = 0.66), time effect (P < 0.001) and treatment by time effect interaction (P = 0.72). (C) Prescapular; Average (10% = 30.1^{b}, 15% = 30.7^{ab}, 20% = 31.9^{a}; SEM 0.6), treatment effect (P = 0.09), time effect (P = 0.11) and treatment by time effect interaction (P = 0.54). (D) Thorax wall; Average (10% = 32.5, 15% = 33.0, 20% = 33.4; SEM 0.45), treatment effect (P = 0.36), time effect (P < 0.001) and treatment by time effect interaction (P = 0.83). (E) Thigh; Average (10% = 30.5, 15% = 30.0, 20% = 30.6; SEM 1.1), treatment effect (P = 0.87), time effect (P < 0.001) and treatment by time effect interaction (P = 0.97). (F) Shin; Average (10% = 26.0, 15% = 27.8, 20% = 27.8; SEM 0.93), treatment effect (P = 0.23), time effect (P < 0.001) and treatment by time effect interaction (P = 0.62). (G) Foot; Average (10% = 23.2, 15% = 26.8, 20% = 24.6; SEM 1.14), treatment effect (P = 0.11), time effect (P < 0.001) and treatment by time effect interaction (P = 0.11). (H) Tail; Average (10% = 19.4, 15% = 22.5, 20% = 20.9; SEM 1.18), treatment effect (P = 0.18), time effect (P < 0.001) and treatment by time effect interaction (P = 0.34).

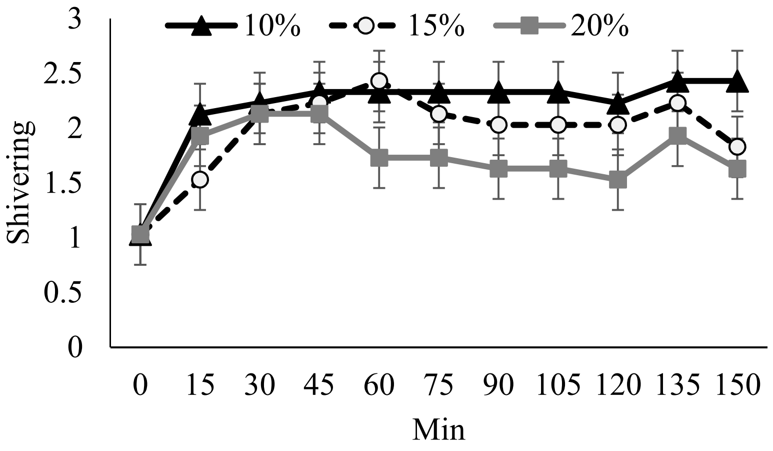

Figure S2 Shivering across time, during a cold challenge of newborn dairy calves fed different volume of colostrum. 10% BW as colostrum (n = 10); 15% BW as colostrum (n = 10); 20% BW as colostrum (n = 10). Average (10% = 2.2^{a}, 15% = 2.0^{ab}, 20% = 1.7^{b}; SEM 0.19), treatment effect ($P = 0.10$), time effect ($P < 0.001$), treatment by time effect interaction ($P = 0.77$).

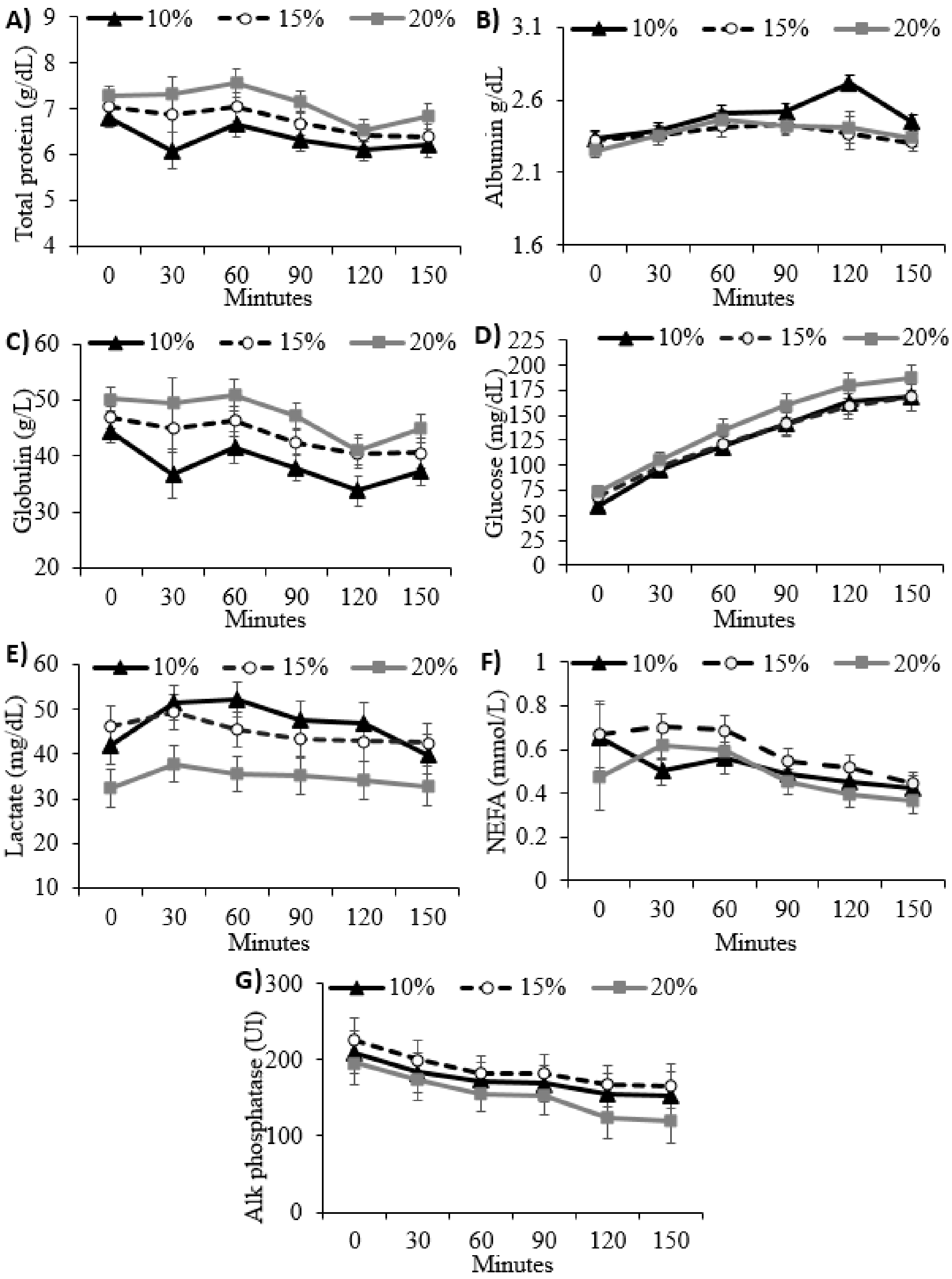

Figure S3 Blood metabolites of newborn dairy calves fed different volume of colostrum, across time effect during a cold challenge. 10% BW as colostrum (n = 10); 15% BW as colostrum (n = 10); 20% BW as colostrum (n = 10). (A) Total protein; Average (10% = 6.4^{b}, 15% = 6.7^{ab}, 20% = 7.1^{a}; SEM 0.23), treatment

effect (P = 0.09), time effect (P < 0.001), treatment by time effect interaction (P = 0.86). (B) Albumin; Average (10% = 2.5^{a}, 15% = 2.4^{b}, 20% = 2.4^{b}; SEM 0.05), linear effect (P = 0.06), quadratic effect (P = 0.21), treatment effect (P = 0.08), time effect (P = 0.01), treatment by time effect interaction (P = 0.48). (C) Globulin; Average (10% = 38.6^{b}, 15% = 43.6^{ab}, 20% = 47.3^{a}; SEM 2.20), treatment effect (P = 0.03), time effect (P < 0.001), treatment by time effect interaction (P = 0.92). (D) Glucose; Average (10% = 125.6, 15% = 127.1, 20% = 141.3; SEM 8.51), treatment effect (P = 0.34), time effect (P < 0.001), treatment by time effect interaction (P = 0.90). (E) Lactate; Average (10% = 46.7^{a}, 15% = 44.9^{a}, 20% = 34.6^{b}; SEM 3.85), treatment effect (P = 0.07), time effect (P = 0.03), treatment by time effect interaction (P = 0.17). (F) NEFA; Average (10% = 0.5, 15% = 0.6, 20% = 0.5; SEM 0.05), treatment effect (P = 0.20), time effect (P < 0.001), treatment by time effect interaction (P = 0.73). (G) Alkaline phosphatase; Average (10% = 174.1, 15% = 187.1, 20% = 153.7; SEM 23.7), treatment effect (P = 0.33), time effect (P < 0.001), treatment by time effect interaction (P = 1.00).

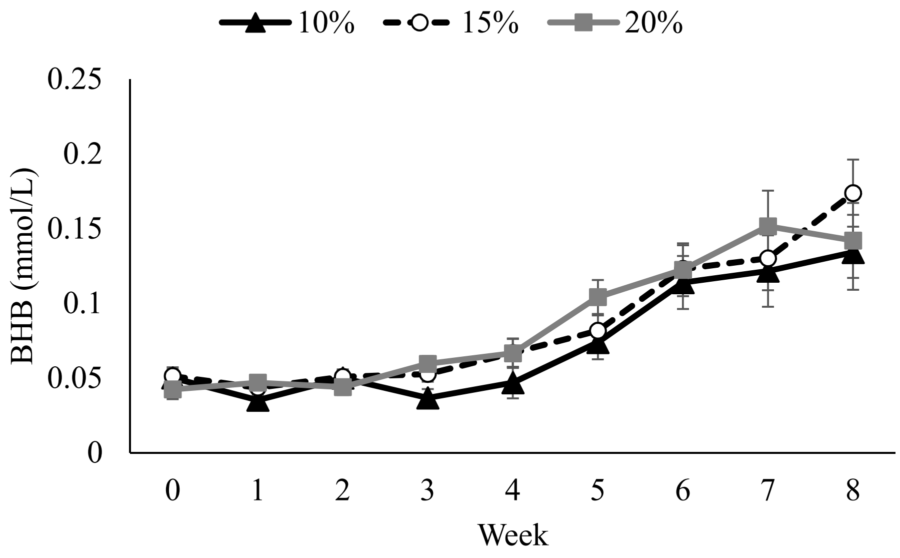

Figure S4 Preweaning beta-hydroxybutyrate concentrations of dairy calves fed different volumes of colostrum at birth. 10% BW as colostrum (n = 10); 15% BW as colostrum (n = 10); 20% BW as colostrum (n = 10). Treatment effect ($P < 0.09$) Age effect ($P < 0.001$) and treatment by age interaction ($P = 0.50$).
